# Study of the Spatio-Temporal Differentiation of Factors Influencing Carbon Emission of the Planting Industry in Arid and Vulnerable Areas in Northwest China

**DOI:** 10.3390/ijerph17010187

**Published:** 2019-12-26

**Authors:** Yujie Huang, Yang Su, Ruiliang Li, Haiqing He, Haiyan Liu, Feng Li, Qin Shu

**Affiliations:** 1 College of Economics and Trade, Xinjiang Agricultural University, Urumqi, Xinjiang 830052, China; 320192793@xjau.edu.cn (Y.H.); 320192791@xjau.edu.cn (Q.S.); 2 College of Letters and Science, University of Wisconsin-Madison, Madison, WI 53706, USA; rli328@wisc.edu; 3 School of Geomatics, East China University of Technology, Nanchang 330013, China; hhq201360010@ecit.cn; 4 School of Water Resources and Environmental Engineering, East China University of Technology, Nanchang 330013, China; hy_liu@ecut.edu.cn; 5 School of business administration, Xinjiang University of Finance and economics, Xinjiang 830012, China; lifeng@xjufe.edu.cn

**Keywords:** Northwest China, vulnerable area, agriculture, carbon emission, inflection point, spatio-temporal differentiation, EKC model

## Abstract

Due to the importance of understanding the relationship between agricultural growth and environmental quality, we analyzed how high-quality agricultural development can affect carbon emissions in Northwest China. Based on the concept of the environmental Kuznets curve, this study uses provincial panel data from 1993 to 2017 to make empirical analyses inflection point changes and spatio-temporal differences in agricultural carbon emissions. The highlights of our findings are as follows: (1) In Northwest China, there is an inverse N-shape curve, and the critical values are 3578 yuan/hm^2^ and 45,738 yuan/hm^2^, respectively. (2) For 2017, the agricultural economic intensity was 50,670 yuan/hm^2^, exceeding the critical value (high inflection point) of 45,738 yuan/hm^2^. (3) Ningxia, Gansu, and Qinghai have not reached the turning point. Having comparable climate, natural conditions, and overall environmental factors, these three provinces would reach the turning point at similar time periods. (4) The average value in agricultural carbon emission intensity in the region is 767.79 kg/hm^2^, and the order based on intensity is Xinjiang > Shaanxi > Ningxia > Gansu > Qinghai.

## 1. Introduction

In order to contribute to lowering global greenhouse gas (GHG) emissions, countries must reduce their carbon footprints, which would require improving their ecological environment and advocating for a low-carbon economy. Tian et al. [1] found that land-use change, agricultural activities, and waste management are the primary sources of terrestrial biogenic greenhouse gases and significantly contribute to climate change through increased CH_4_ and N_2_O emissions. From the sixth Intergovernmental Panel on Climate Change (IPCC) Special Report on Climate Change and Land (2019), greenhouse gases emitted by the agriculture, forestry, and other land-use (AFOLU) sector account for 23% of the total anthropogenic greenhouse gases, while about a third of the natural carbon dioxide absorbed by land is caused by emissions from fossil fuel use and production [2]. Due to the declining vegetation cover, increasing food consumption, and rising energy usage, the amount of GHG in the atmosphere has been increasing exponentially, trapping heat and causing global warming, which further aggravates climate change. Considered as a principal contributor to global carbon dioxide emissions, agriculture, one of China’s primary industries, is closely linked with the problem of greenhouse gas emissions. The resulting ecological crisis would be catastrophic and irreversible, and therefore controlling agricultural carbon emissions has become one of China’s primary concerns.

Since 2008, the concept of “low-carbon economy with low emission and high income” has become widely popular in China. The concept of low-carbon agricultural economy pertains to agricultural production and operations achieving minimal greenhouse gas emissions while achieving desired benefits. Through continuous development, low-carbon agriculture has become an emerging paradigm in agricultural sustainable development, which takes into account economic, ecological, and social benefits in the context of global climate change and low-carbon economic development [3,4,5,6,7]. In China, following the “two-step” trend in the global energy transformation, the agricultural sector is being developed to transition from high carbon to low carbon, and eventually towards becoming carbon-neutral. Since instituting economic reforms, agricultural production conditions have greatly improved, production efficiency has been significantly enhanced, and the production scale has substantially increased. However, alongside rapid development, emissions of carbon dioxide and other GHG from agricultural activities have also increased. Thus, promoting low-carbon agriculture has become an urgent priority, particularly for developing countries, in order to reduce the sector’s carbon footprints without sacrificing agronomic production [3,4,5,6,7,8,9,10,11,12].

As a critical base for animal husbandry, crop production, and characteristic agriculture in China, Northwest China is facing increased ecological pressure to boost the agricultural economy and develop its land resources. However, due to its partly arid and partly semi-arid climate, the water resource in Northwest China is in short supply, and its ecosystem is fragile. Once damaged, the ecosystem degradation is irreversible, threatening the local population’s natural and economic environment. Therefore, research on agricultural carbon emissions, particularly in the arid and vulnerable areas of Northwest China, is of great importance. This could be used to determine the turning point in agricultural economic development where a high emission transitions into low emissions, to help reduce the agricultural carbon footprint and support achieving a high-quality development.

For this study, we analyzed the inflection points and spatio-temporal differences of agricultural carbon emissions in Northwest China to explore the effects of economic growth on carbon emissions and identify significant determinants. Our findings would provide theoretical guidance in support of reducing agricultural carbon emissions and promoting sustainable development of low-carbon agriculture. Compared with the existing research [12,13,14,15,16,17], the innovation of this study lies mainly in three key aspects:

First, our research examined the carbon emission and agricultural economic intensities for agriculture (crop production) in Northwest China. With improvements in agricultural modernization, mechanization and chemical use have become widely utilized in farm operations, which have resulted in increased agricultural yield. As a result, agriculture has become one of the primary sources of greenhouse gas emissions. In order to fully examine agricultural carbon emissions, agricultural carbon sources have to be first identified in order to systematically calculate their carbon emission contributions. Previous studies have analyzed agricultural carbon emissions mainly from a singular perspective. Li and Li [18] measured carbon emissions caused by the configuration of agricultural energy consumption through seven types of carbon sources, such as coal, gasoline, diesel natural gas, and kerosene. Tian and Jiang [19] studied the agricultural carbon emissions in the Hubei Province from the carbon sources, such as chemical fertilizer, pesticide, agricultural film, and diesel. Wang and Sun [20] and Li [21] investigated plowing and irrigation and calculated the total agricultural carbon emissions from the aspects of energy input and land use [18,19,20,21]. Using extensive datasets of cities in Northwest China, this study reevaluated the agricultural carbon emissions to provide more accurate estimates. Second, recent research has mainly focused on carbon emissions from China’s more developed provinces, such as measuring low-carbon agricultural productivity in central and eastern regions and analyzing the dynamic change and evolution trend of agricultural carbon emissions in China’s coastal cities at the provincial level. However, the unique agricultural landscape, environmental constraints, and ecological vulnerability in Northwest China have been overlooked [22,23,24,25,26,27,28,29,30,31,32,33,34,35,36,37,38,39,40,41]. The development of agricultural carbon emissions is dynamic and complex, and studying the carbon emissions for this region is of considerable significance and essential in the construction of the ecological civilization and the development of a regional agricultural economy. Finally, while a number of studies have been conducted with regard to the relationship between the intensity of agricultural carbon emissions and per capita GDP, limited research has been done on the relationship between agricultural carbon emission and agricultural economic growth for Northwest China and the examination of the environmental Kuznets curve (EKC) of agricultural carbon emission [9,42]. Therefore, this study adopted various modifications to traditional carbon estimation approaches, such as using the planting area of crops as starting point, employing agricultural carbon emission intensity to indicate the level of agricultural carbon emission, and agricultural economic intensity to express the level of agricultural economic development. By using such an approach, we were able to clearly distinguish the carbon emissions generated by cropping and other agricultural activities.

## 2. Materials and Method

### 2.1. Research Area

**Figure 1:**
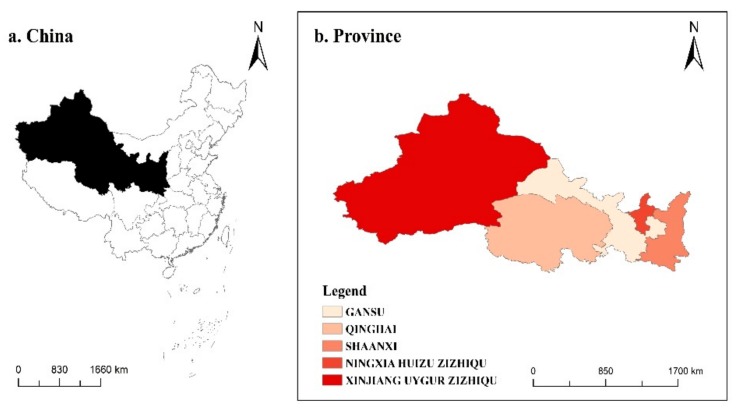
Map of five provinces in Northwest China.

Northwest China is composed of Shaanxi, Gansu, Ningxia, Qinghai, and Xinjiang and serves as an essential trade route, such as the “Silk Road Economic Belt” and “Eurasian land bridge”. The region enjoys unique advantages in natural resources and the agricultural planting industry, which provide a solid economic foundation for the region [27]. The study area can be seen in Figure 1.

By the end of 2018, the permanent population was 102.79 million, with 47.39% of the population engaged in agricultural work. The region has a total area of 3.0456 million square kilometers, accounting for 57.7% of the total area of the western region and 31.7% of the national land area. It consists of 18.53 million hectares of cultivated land and has 0.21 hectares of cultivated land per capita, which is double the national average. Agriculture in Northwest China is based mainly on large-scale planting of cash crops and vegetables. Each province has particular advantages in the agricultural industry, different from other provinces, and the region, in general, has been experiencing considerable agricultural economic growth [28,29].

Northwest China is located in the hinterland of the Eurasian continent, which has very little precipitation and receives no rainfall all year round. The region, which belongs to the arid and semi-arid area, receives annual precipitation of less than 500 mm. The annual precipitation in the Loess Plateau is between 300–500 mm, in Qaidam Basin below 200 mm, in Hexi Corridor less than 100 mm, in Dunhuang about 29.5 mm, in Turpan less than 20 mm, and in Ruoqiang about 10.9 mm. Once the ecosystem is destroyed, environmental degradation becomes irreversible, creating a ‘barrier’ that constrains applications and limits the use [23,24,25,26,27,28,29].

### 2.2. Indicator Selection

The primary agricultural products in Northwest China are vegetables and corn. Aside from these crops, other agricultural products could be found in particular areas of the region. When differentiated by province, the dominant agricultural products for Shaanxi are vegetables, corn, and soybeans; for Gansu, corn, vegetables, and wheat; for Qinghai, corn, vegetables, and rice; for Ningxia, cotton and vegetables; and, for Xinjiang, corn.

Agricultural greenhouse gases in Northwest China come from two primary sources: nitrous oxide discharges from crop production and carbon emissions from farm activities. In the process of crop planting, the destruction of soil surface causes a large volume of N_2_O gas to be released in the atmosphere. N_2_O can be characterized as having high-temperature potential, long retention time, and can cause damage to the ozone layer. In this study, the N_2_O emission coefficients of rice, spring wheat, winter wheat, soybean, corn, vegetable, cotton, and other crops, were based on previous studies [36,37,38,39,40,41]. In order to compare values, the N_2_O emissions were converted into their C equivalent using the conversion method 1t N_2_O = 81.27 t C. For carbon emissions from farming activities, the quantity can be determined using aggregated values of the following: (1) carbon emissions directly or indirectly generated by the application of chemical fertilizer; (2) carbon emissions directly or indirectly generated by the use of pesticides; (3) carbon emissions directly or indirectly generated by the use of agricultural plastic film; (4) carbon emissions generated by the use of agricultural diesel oil consumed by agricultural machinery; (6) carbon emissions generated by the loss of soil organic carbon pool caused by the conversion of agricultural lands; and, (6) carbon emissions generated by the consumption of electric energy in the process of agricultural irrigation.

In order to avoid the variations in the agricultural production scale, we selected intensity indices to measure agricultural economic growth and environmental quality. The index of agricultural economic growth is P-AGDP (per unit sown area Agricultural GDP), which is equal to the gross agricultural product per unit sown area. The total agricultural output value and planting area of crops over the years were derived from the statistical yearbook of each province published on the website of the China Statistics Bureau.

(1) P−AGDP=gross output value of agriculture/Planting area of crops.

There may be a more direct causal relationship between environmental quality and economic growth present. In the initial stage of agricultural development, growth mainly depends on increasing labor input. The range of change in agricultural material input is relatively small, and the carbon emission intensity per unit sown area may have mixed effects (increase, decrease, or stay the same). With the maturation of the agricultural sector, the output contribution of labor gradually weakens, and agricultural growth begins to rely more on fertilizer use. An increase in farm material inputs (e.g., pesticides) results in improved agricultural yield but can also intensify carbon emissions. When agriculture develops to a certain level, in order to avoid continued deterioration of the ecological environment, advanced technology will be widely used for agricultural production, and the dependence on farming materials will be gradually reduced. At this stage, the farm output will continue to grow, while the farm carbon emissions will continue to increase.

### 2.3. Agricultural Carbon Emission Estimation

At present, most studies have made use of agricultural carbon emission intensity (PCO_2_) in studying environmental quality indicators. Based on the agrarian characteristics of the region, we focused our estimation on the carbon emissions by the following sources: chemical fertilizer use, pesticide application, use of agricultural film and other agricultural materials, fuel consumption by farm machinery, carbon released caused by plowing, and electric consumption by irrigation activities (Table 1). During crop planting, tilling and other soil disturbing activities cause massive amounts of greenhouse gases to be released in the atmosphere, which further compounds the problem of GHG emissions (Table 2) [31]. The sown area for each crop type was derived from the *Statistical Yearbook* published by the China Statistics Bureau website. The planting area of each crop comes from the Statistical Yearbook published on the website of the China Statistics Bureau. The intensity of agricultural carbon emission is obtained by dividing the total amount of agricultural (planting) carbon emission by the planting area of crops [32,33]. The agrarian carbon emission was then estimated using the equation:

(2) $$C=\sum C_{it}=\sum T_{it}\times \alpha_{i},$$

where *C* is the total amount of agricultural carbon emissions; *C_it_* is the agrarian carbon emissions in year *t* of type *i* carbon source; *T_it_* is the amount of carbon source in year *t* of type *i;* and *α_i_* is the carbon emission coefficient for each carbon source type *i*. Considering the different chemical properties of N_2_O and C, calculating them directly would be difficult. In this paper, N_2_O emissions were converted into equivalent C using the conversion rate: 1 t N_2_O = 81.27 t C.

### 2.4. Environmental Kuznets Curve

Since its establishment, the environmental Kuznets curve (EKC) has been used to measure the deterioration of environmental quality with economic growth in the early stage of economic development. When the economy develops into a particular stage, the environmental quality will improve with economic growth. With the development of China’s agriculture, a number of challenges, such as less arable land per capita, unskilled agricultural employees, low level of agricultural science and technology, and wastage in agricultural production resources and energy, have exacerbated the problem of high carbon emissions. In recent decades, with China’s high levels of agricultural and economic development, people have started paying more attention on how to control its agricultural carbon emissions and minimize its impact on the global environment. A number of theoretical and empirical research has been conducted on the relationship between carbon emissions and economic growth, and many regression models have been proposed. In constructing the model, we chose a relatively flexible cubic polynomial form, where the results can be monotone linear, inverted U-shaped, or N-shaped (Table 3) [43].

Where *E_t_* is the environmental pressure of the country or region at time *t*; *Y_t_* is the economic output at time *t*; and *β*_1_, *β*_2_, *β*_3_ are the parameters to be estimated. *E_t_* is commonly expressed by ecological quality indicators (e.g., pollutant emission intensity). In this study, we used agricultural carbon emission intensity (*PCO_2_*) to denote environmental quality and agricultural economic intensity (*P-AGDP*) to indicate economic output [44,45,46,47,48,49].

The estimation form of the cubic polynomial of the EKC curve was used to construct the model. In order to reduce the fluctuation and eliminate possible heteroscedasticity in the data, the natural logarithm of agricultural carbon emission and production per unit planting area were used were recorded as lnPCO_2t_ = ln (PCO_2_) and lnP-AGDP_t_ = ln (P-AGDP). In this study, the theoretical model of Grossman and Krueger was used to provide the relationship between agricultural carbon emission intensity and agricultural economic intensity [50,51]:

(3) $$\ln PCO_{2t}=\beta_{0}+\beta_{1}\ln{\left(P-AGDP\right)}_{t}+\beta_{2}\ln{\left(P-AGDP\right)}_{t}^{2}+\beta_{3}\ln{\left(P-AGDP\right)}_{t}^{3}+\varepsilon_{t},$$

where *PCO_2t_* is the intensity value of agricultural carbon emission in year *t*; *P-AGDP_t_* is the intensity value of agrarian economy in year *t*; *ε_t_* is the random error term; *β_0_* is the intercept; and *β*_1_, *β*_2_, and *β*_3_ are the parameters to be estimated.

### 2.5. Data Collection

The data used in this study were derived from *China Statistical Yearbook, Xinjiang Statistical Yearbook, Shaanxi Statistical Yearbook, Ningxia Statistical Yearbook*, Gansu Statistical Yearbook, and *Qinghai Statistical Yearbook*. The following spatial data were gathered for each of the five provinces of Northwest China: rice planting area (hm^2^), spring wheat planting area (hm^2^), winter wheat planting area (hm^2^), soybean planting area (hm^2^), corn planting area (hm^2^), vegetable planting area (hm^2^), cotton planting area (hm^2^), total crop planting area (kg), effective irrigation area (hm^2^), and actual crop planting area. Additionally, the following farm-related data were also collected: the net amount of agricultural fertilizer (kg), the use of pesticide (kg), the use of agricultural plastic film (kg), and the agricultural diesel oil (kg) consumed for agricultural activities. In order to eliminate the impact of price fluctuations, the total agricultural output values were adjusted to 1993 prices. The time series of China’s agricultural economic intensity in 1993–2017 can be obtained by dividing the two [9,23].

## 3. Results

### 3.1. Augmented Dickey-Fuller (ADF) Unit Root Test

The natural logarithm sequences lnPCO_2_, lnP-AGDP, (lnP-AGDP)^2^, and (lnP-AGDP)^3^, as well as their first-order difference sequences, were examined using the unit root test. As shown in Table 4, the natural logarithm sequences lnPCO_2_, lnP-AGDP, (lnP-AGDP)^2^, and (lnP-AGDP)^3^ were all non-stationary, while the first-order difference sequences lnPCO_2_ (1), lnP-AGDP (1), (lnP-AGDP)^2^ (1), and (lnP-AGDP)^3^ (1) are stationary. Therefore, the lnPCO_2_ (1), lnP-AGDP (1), (lnP-AGDP)^2^ (1), and (lnP-AGDP)^3^ (1) values meet the conditions of the cointegration test.

### 3.2. Johansen Cointegration Test

The cointegration test showed that the lnPCO_2_ (1), lnP-AGDP (1), (lnP-AGDP)^2^ (1), and (lnP-AGDP)^3^ (1) rejected the hypothesis, indicating no cointegration relationship between the variables at 5% significance level. As shown in Table 5, there was at least one cointegration vector between the lnPCO_2_, lnP-AGDP, (lnP-AGDP)^2^, (lnP-AGDP)^3^ sequences with a long-term equilibrium relationship, and there was no pseudo-regression problem.

### 3.3. Granger Causality Test

As shown in Table 6, for the level 3 delay, the original hypothesis, “lnP-AGDP is not Granger cause of lnPCO_2_”, can be rejected at the 10% confidence level. For level 1 delay, the initial hypothesis, “lnPCO_2_ is not Granger cause of lnP-AGDP”, can be dismissed at the 5% confidence level. The results suggest a two-way Granger causality between lnPCO_2_ and lnP-AGDP, which indicates that agricultural economic growth affects the change in agricultural carbon emissions to a certain extent and that the changes in carbon emissions also impact agricultural economic growth. The existence of causality ensures that the follow-up regression analysis and EKC verification results are accurate and reliable.

### 3.4. EKC Test and Inflection Point Analysis

Through the linear regression of lnPCO_2_ and lnP-AGDP, the Durbin-Watson (D–W) statistic of the model was small, indicating the presence of autocorrelation in the regression residual. Therefore, autocorrelation (AR) (1) and AR (2) were added to the regression equation. The modified D–W statistic of the sample was 2.0248, eliminating the autocorrelation. Therefore, the third regression model (model 9) had the best fitting effect, and all variables passed the significance test. Finally, the regression equation is obtained as follows:

(4) $$\ln PCO_{2t}=768.2418-98.8143\ln{\left(P-AGDP\right)}_{t}+4.2588{\left(lnP-AGDP\right)}_{t}^{2}-0.0610{\left(lnP-AGDP\right)}_{t}^{3}+\varepsilon_{t}.$$

According to Table 7, the regression equation satisfied the conditions β_1_ < 0, β_2_ > 0, and β_3_ < 0 indicates an inverted N-shaped relationship between the intensity of agricultural carbon emission and the intensity of the agricultural economy. However, determining whether there is an inflection point with an inverted N-shaped EKC requires further calculation. For the EKC curve, the extreme point can be obtained by the derivative. The derivation of model 9 is as follows:

(5) $$\ln PCO_{2t}=-98.8143+8.5176\ln{\left(P-AGDP\right)}_{t}-0.183\ln{\left(P-AGDP\right)}_{t}^{2}+\varepsilon_{t},$$

△ = (8.5176)^2−4 × (−0.183) × (−98.8143) = 0.2174 > 0.(6)

Through calculations, we can find the two extreme points in the equation. By letting(lnPCO_2t_) = 0, we get [lnP-AGDPt]1 = 21.9981 and [lnP-AGDPt]2 = 24.5462. Due to the coefficient of (lnP-AGDPt)3 = −0.0610 < 0, the smaller real root (21.9981) is the minimum point, while the larger real root (24.5462) is the maximum point. We also found that (P-AGDP_t_)1 = 3578 and (P-AGDP_t_)2 = 45738, indicating that the inflection points are 3578 yuan/hm^2^ and 45,738 yuan/hm^2^.

## 4. Discussion

### 4.1. Economic Implications of the Inflection Point Analysis

When the agricultural output per unit sown area is less than 3578 yuan/hm^2^, the agricultural carbon emission intensity and the agricultural economic intensity show a reverse relationship, such that the agricultural economy would keep on growing, and the agricultural carbon emission intensity would show a downward trend. In this stage, the increase in agricultural output may be more dependent on labor because the agricultural material input has not increased dramatically. Furthermore, the progress in agricultural technology has improved the utilization efficiency of agricultural materials to a certain extent, thus reducing the intensity of agricultural carbon emission per unit sowing area.

When the agricultural economic intensity is between 3578 yuan/hm^2^ and 45,738 yuan/hm^2^, the agricultural carbon emission and the agricultural economic development have a synchronous ascending trend. The rise in the total agricultural output value per unit planting area would mean an increase in the agricultural carbon emission intensity. With the gradual weakening of labor’s contribution to agricultural output, in this stage, the development of the agricultural economy depends more on agricultural material inputs, such as chemical fertilizer, pesticide, agricultural film, and agricultural machinery. While realizing rapid improvements in agricultural economic strength, carbon emissions from farms also rise at similar rates.

When the intensity of the agricultural economy exceeds 45,738 yuan/hm^2^, the carbon emission intensity gradually decreases with the growth of the agricultural economy. At this stage, people begin to realize that even if high inputs in the agricultural development model can result in rapid economic growth, the damage to the ecological environment, particularly on soil resources, could not be overlooked. Therefore, advanced production technologies and concepts become widely used in agricultural production, such as cultivating enhanced varieties, improving the utilization rate of agricultural materials, developing facility agriculture and circular agriculture, and accelerating the pace of agricultural modernization. In doing so, the agricultural economy maintains a sustained growth trend while the intensity of agricultural carbon emission gradually decreases.

### 4.2. Spatio-Temporal Differentiation of Inflection Point

In 2017, the intensity of the agricultural economy was 50,670 yuan/hm^2^, which exceeded the critical value (45,738 yuan/hm^2^). This means that with further development of the agricultural economy, the intensity of agricultural carbon emission will show a downward trend. In terms of spatial distribution, we compared the agricultural economic intensity per unit sown area for 2017 with the critical value of 45,738 yuan. We found that the agricultural economic intensity in Shaanxi (74,406 yuan) and Xinjiang (52,224 yuan) exceeded the inflection point value, which suggests that the agricultural carbon emission level in these areas will gradually reduce with the development of the agricultural economy. Gansu, Ningxia, and Qinghai were found to have lower values than the inflection point of agricultural carbon emissions.

For the time path difference, we estimated the time point by determining the average annual growth rate of the actual agricultural economic strength [52]. We calculated the annual growth rate of the agricultural economic intensity for each province from 1993 to 2017. For each province, the annual growth rate of the estimated time point was taken, which was then used to calculate and determine the number of years required to reach the inflection point value. The three provinces (i.e., Ningxia, Gansu, and Qinghai) that have not reached the inflection points were further examined, and the summary of results is shown in Table 8. For the three provinces, the number of years needed for the carbon emission to reach the inflection point of the EKC curve was relatively similar, mainly because these provinces comprise the national economic crop planting areas. For Ningxia, a year delay in reaching the inflection point compared with Gansu and Qinghai could be the result of its planting industry being behind the other provinces. Moreover, constraints in climate and environmental conditions could have contributed to the lower output level in Ningxia.

## 5. Conclusions

Previous studies have focused mainly on agricultural carbon emissions for whole countries or regions with highly developed agricultural sectors [53,54]. Research on agricultural carbon emissions and agricultural output in arid and ecologically fragile areas such as Northwest China has remained limited. In addition, the per capita indicators that have commonly been used to measure economic growth and environmental quality, result in either overestimation or underestimation. Since the exact number of people cannot be accurately determined, it would be difficult to classify and define the labor force in crop production, forestry, animal husbandry, and aquaculture. A limited number of studies have also used the EKC curve test results of agricultural carbon emissions and examined the time inflection points. In this study, we made improvements in the current literature, which can be summarized as follows:

1. As an important base in animal husbandry, crop production, and prairie in China, the implementation of the “western development” strategy has accelerated the development of the agricultural economy in Northwest China. This has resulted in increased carbon emissions that threaten the region’s ecological balance. Due to its arid and semi-arid climate, once the ecosystem in Northwest China is destroyed, it will be irreversible. Therefore, it is critical to the local economy and the environment to examine how to ensure that economic growth will not contribute to increased carbon emissions;
2. The research focused on emission intensity from using agricultural materials and soil carbon emissions. Livestock breeding was not incorporated as part of the study and focused solely on the planting industry. The planting area of crops was used as the starting point, and the carbon emission and agricultural economic intensities were used to express the agricultural carbon emission level and agricultural economic development level;
3. There was an inverse N-shaped EKC relationship between agricultural carbon emission intensity and agricultural economic intensity, with critical values at 3578 yuan/hm^2^ and 45,738 yuan/hm^2^. Its economic meaning is that when the agricultural output per unit sown area reaches 3578 yuan/hm^2^, the intensity of agricultural carbon emissions starts to rise from the decline; when the agricultural economic intensity exceeds 45,738 yuan/hm^2^, the intensity of agricultural carbon emissions will gradually decrease; when the agricultural economic intensity is between the critical value of the double turning point, the two are in a synchronous rising trend;
4. The agricultural economic intensity for 2017 is 50,670 yuan/hm^2^, exceeding the critical value of the high inflection point (45,738 yuan/hm^2^). This suggests further economic growth will result in a downward trend in carbon emission intensity. The agricultural economic intensities of Shaanxi and Xinjiang exceed the inflection point value, which are the main production areas for crop production. Compared with Gansu, Ningxia, and Qinghai, they have a large agricultural population and have a more developed agricultural economy and technology;
5. Three provinces (Ningxia, Gansu, and Qinghai) that have not reached the inflection point were further analyzed. The inflection point of agricultural carbon emissions showed clear correlation. For these three provinces, the years needed to reach the inflection point were similar with similar climate, natural conditions, and overall environmental factors. The primary difference could be found in the smaller rural population in Ningxia, resulting in lower output levels from a smaller planting industry. This could have affected the lag in agricultural economic growth, which could explain the slight time delay to reach the turning point, compared to Gansu and Qinghai.

Northwest China, as an economically backward and environmentally fragile region, is dominated by an agricultural economy. This study found that the average value of agricultural carbon emission intensity in the five northwestern provinces is 769.79 kg/hm^2^, and the order of agricultural carbon emission intensity is Xinjiang > Shaanxi > Ningxia > Gansu > Qinghai. In order to bring down emission levels, the government and the local population, particularly the farmers, ought to focus on advocating sustainable development of low-carbon agriculture. In agricultural production, we can promote scientific and technological progress and institutional innovation in combination with government policies, such as strengthening the application of remote sensing technology in the field of agriculture, improving the input structure of agricultural production factors in terms of soil, water resources, prevention and control of agricultural diseases and insect pests, reducing agricultural (planting) pollution, and improving agricultural economic benefits [55,56,57,58,59]. Areas with inverted N-shaped EKC should prepare and formulate necessary steps to avoid joint growth of its agricultural economy and its carbon emissions.

## Figures and Tables

**Table 1:** Emission coefficients of main carbon sources.

Carbon Source	Carbon Emission Coefficient	Reference Source
Chemical fertilizer	0.8956 kg C/kg	T.O.WEST [32] Oak Ridge National Laboratory
Pesticides	4.9341 kg C/kg	Oak Ridge National Laboratory [33]
Agricultural film	5.18 kg C/kg	Institute of agricultural resources and ecological environment, Nanjing Agricultural University
Diesel oil	0.5927 kg C/kg	IPCC
Plowing	312.6 kg C/km^2^	School of Biology and Technology, China Agricultural University [34]
Irrigation	266.48 kg C/hm^2^	Duan et al. [35]

**Table 2:** Emission coefficient of various crop varieties.

Crop Varieties	N_2_O Emission Coefficient/(kg/hm^2^)	Reference Source
Unhusked rice	0.24	Wang [36]
Spring wheat	0.4	Yu et al. [37]
Winter wheat	2.05	Pang et al. [38]
Soybean	0.77	Xiong et al. [39]
Corn	2.532	Wang et al. [40]
Vegetables	4.21	Qiu et al. [41]
Cotton	0.4804	Wang [36]

Note: 1 t N_2_O = 81.27 t C.

**Table 3:** Regression models analyzed in the study.

**Linear**
E_t_ = α + β_1_Y_t_ + ε_t_
lnPCO_2_ = β_0_ + β_1_ln(P-AGDP)_t_ + ε_t_
where β_1_ > 0
Non-Linear. **Quadratic Model**
E_t_ = α + β_1_Y_t_ + β_2_Y_t_ + ε_t_
lnPCO_2_ = β_0_ + β_1_ln(P-AGDP)_t_ + β_2_ln(P-AGDP)_t_^2^ + ε_t_
where β_2_ < 0 or β_2_ > 0
**Cubic Model**
Et = α + β_1_Y_t_ + β_2_Y_t_^2^ + β_3_Y_t_^3^ + ε_t_
lnPCO_2_ = β_0_ + β_1_ln(P-AGDP)_t_ + β_2_ln(P-AGDP)_t_^2^ + β_3_ln(P-AGDP)_t_^3^ + ε_t_
where β_3_ > 0

Note: ε is a random error term.

**Table 4:** Unit root test results.

Sequence	LnPCO_2_	LnPCO_2_ (1)	LnP-AGDP	LnP-AGDP (1)	(lnP-AGDP)^2^	(lnP-AGDP)^2^ (1)	(lnP-AGDP)^3^	(lnP-AGDP)^3^ (1)
ADF test value	5.5688	−4.7912	2.3659	−7.0151	2.4519	−6.8869	2.5448	−6.7404
Prob.	1.0000	0.0009	0.9939	0.0000	0.995	0.0000	0.9959	0.0000
1% critical value	−2.6649	−3.7529	−2.6649	−3.7529	−2.6649	−3.7529	−2.6649	−3.7529
5% critical value	−1.9557	−2.9981	−1.9557	−2.9981	−1.9557	−2.9981	−1.9557	−2.9981
10% critical value	−1.6088	−2.6388	−1.6088	−2.6388	−1.6088	−2.6388	−1.6088	−2.6388
Conclusion	Nonstationary	stable	Nonstationary	stable	Nonstationary	stable	Nonstationary	stable

Note: () indicates lag order.

**Table 5:** Cointegration test results of variables.

Original Hypothesis	With 0 Cointegration Vectors	With at Least 1 Cointegration Vector
characteristic value	0.5270	0.3531
Trace statistics	27.2373	10.0166
5% critical value	20.2618	9.1645
*p*-value	0.0046	0.0344

**Table 6:** Causality test results based on different Lag Length.

Index	LnP-AGDP Is Not lnPCO_2_ Granger Cause LnP-AGDP Does Not Granger Cause lnPCO_2_		lnPCO_2_ Is Not lnP-AGDP Granger Cause lnPCO_2_ Does Not Granger Cause lnP-AGDP	
	F-Statistic	Prob.	F-Statistic	Prob.
1	2.1531	0.1571	4.9635	0.037
2	1.5017	0.2494	1.0371	0.3747
3	3.2067	0.0535	1.6427	0.2218

Note: P-AGDP is the average GDP.

**Table 7:** Carbon emission model in Northwest China.

Index	Linear (1)	Linear (2)	Linear (3)	Quadratic (4)	Quadratic (5)	Quadratic (6)	Cubic (7)	Cubic (8)	Cubic (9)
Ln(P-AGDP)	0.2713	0.2593	1.9916	−0.0956	−1.1367	−1.1124	−67.5346	−95.9554	−98.8143
[LN(P-AGDP)]^2^				0.0079	0.0273	0.0261	2.9313	4.1360	4.2588
[LN(P-AGDP)]^3^							−0.0422	−0.0592	−0.0610
C	0.3142	0.59	5.4195	4.5728	18.24	18.3158	522.7035	746.0761	768.2418
AR (1)		0.3052	1.4441		0.9824	1.3944		0.8435	0.9751
AR (2)			−0.4551			−0.4138			−0.1665
Model test and summary statistics									
R^2^	0.9553	0.9578	0.9811	0.9559	0.9806	0.983	0.9682	0.9866	0.9869
P	0.0000	0.0000	0.0000	0.0000	0.0000	0.0000	0.0000	0.0000	0.0000
D–W	1.5237	1.9916	2.35	1.3934	1.5332	2.2684	0.8806	1.7036	2.0248
Assume the shape of EKC curve when it exists	Monotonous rise	Monotonous rise	Monotonous rise	Type U	Type U	Type U	Inverted N-type	Inverted N-type	Inverted N-type

Note: lnPCO_2_ and lnP-AGDP are the original data, and P-AGDP is the local average GDP.

**Table 8:** Summary of values for Ningxia, Gansu, and Qinghai detailing the time required to reach the inflection point.

Index	Ningxia	Gansu	Qinghai
Current value (2017)	35,347.81	38,552.59	38,665.79
Current annual growth rate (%)	11.66	11.67	11.61
Years to reach inflection point	1.16	0.94	0.94
Specific year of reaching the inflection point	2020	2019	2019
